# ASO Author Reflections: Comparison of Hospital Volume and Risk-Standardized Mortality Rate as a Proxy for Hospital Quality in Complex Oncologic Hepatopancreatobiliary Surgery

**DOI:** 10.1245/s10434-024-15441-3

**Published:** 2024-08-13

**Authors:** William T. Julian, Lee M. Ocuin

**Affiliations:** 1 Case Western Reserve University School of Medicine, Cleveland, OH USA; 2 Division of Surgical Oncology, Department of Surgery, University Hospitals Cleveland Medical Center, Cleveland, OH USA

## Past

The high morbidity and mortality associated with oncologic hepatopancreatobiliary (HPB) surgery has drawn attention to the heterogeneity of outcomes across centers, and centralization to higher-volume centers has been proposed.^1^ The relationship between hospital volume and surgical outcomes has been well established, but patient redistribution and centralization remain controversial, both in scale and scope.^2^ While centralization efforts in Europe and Canada have been met with a decrease in overall mortality, the feasibility of such initiatives in the United States (US) is contentious due to logistical constraints to patients and hospitals, as well as the diverse geographic and insurance landscapes throughout the US.^3^ Additionally, population and case mix differences have made direct comparisons of hospital quality difficult. Risk-standardized mortality rates (RSMR), which can adjust for differences in patient populations, procedure mix, and case volume, have been adopted as another proxy of hospital quality. Currently, no comparison of surgical volume and RSMR exists for oncologic HPB surgery in the US, particularly in the context of patient redistribution.

## Present

Recent studies have indicated that RSMR may outperform hospital surgical volume as a proxy for overall hospital quality. A 2016 study of patients undergoing oncologic surgery in Germany concluded that RSMR outperformed hospital volume in reducing patient mortality and minimizing the logistic burden.^4^ However, given geographical, structural, and institutional differences between Germany and the US, the applicability of their findings, particularly to high-morbidity and mortality HPB surgical procedures, is unclear.

Comparison of RSMR and surgical volume throughout the US for patients undergoing surgery for liver, biliary tract cancer (BTC), and pancreas cancer demonstrated that both high-volume centers (HVCs) and high-performance centers designated by RSMR had the lowest rates of patient mortality at 90 days for all types of cancers studied. However, stratification by volume demonstrated heterogeneity of outcomes, with approximately 15–20% of HVCs not classified as high-performance centers, and approximately 30% of low-volume centers and 45% of medium-volume centers classified as high-performance centers by RSMR. Additionally, simulated redistribution within US census regions demonstrated that an RSMR-based approach more effectively reduced mortality, with a higher reduction in mortality per patient moved compared with volume-based redistribution, while redistributing patients to more centers.^5^ These data suggest that RSMR may be a better proxy for hospital quality than hospital volume alone. Importantly, patient demographics and case mixes were comparable across volume and RSMR stratifications, suggesting that facility-specific factors rather than patient selection may be more important in patient outcomes.

## Future

Discussions about patient redistribution both in the context of patient mortality and cost savings continue. Currently, limitations of our study and other studies include the inability to accurately calculate the logistical constraints imposed by redistribution. Future studies must have access to geographic information of both the patient and treating facility to adequately assess the impact of the logistic burden associated with possible patient redistribution. Furthermore, facility-specific factors contributing to high-performance status should be investigated, which could be generalized to other hospitals and help offset the need for redistribution.
